# Large mass-independent sulphur isotope anomalies link stratospheric volcanism to the Late Ordovician mass extinction

**DOI:** 10.1038/s41467-020-16228-2

**Published:** 2020-05-08

**Authors:** Dongping Hu, Menghan Li, Xiaolin Zhang, Alexandra V. Turchyn, Yizhe Gong, Yanan Shen

**Affiliations:** 1 School of Earth and Space Sciences, University of Science and Technology of China, Hefei, 230026 China; 2 Department of Earth Sciences, University of Cambridge, Cambridge, CB2 3EQ UK

**Keywords:** Biogeochemistry, Geochemistry, Sedimentology

## Abstract

Volcanic eruptions are thought to be a key driver of rapid climate perturbations over geological time, such as global cooling, global warming, and changes in ocean chemistry. However, identification of stratospheric volcanic eruptions in the geological record and their causal link to the mass extinction events during the past 540 million years remains challenging. Here we report unexpected, large mass-independent sulphur isotopic compositions of pyrite with Δ^33^S of up to 0.91‰ in Late Ordovician sedimentary rocks from South China. The magnitude of the Δ^33^S is similar to that discovered in ice core sulphate originating from stratospheric volcanism. The coincidence between the large Δ^33^S and the first pulse of the Late Ordovician mass extinction about 445 million years ago suggests that stratospheric volcanic eruptions may have contributed to synergetic environmental deteriorations such as prolonged climatic perturbations and oceanic anoxia, related to the mass extinction.

## Abstract (web summary)

Identification of stratospheric volcanic eruptions in the geological record and their link to mass extinction events during the past 540 million years remains challenging. Here, the authors report unexpected, large mass-independent sulphur isotopic compositions of pyrite in Late Ordovician sedimentary rocks, which they suggest originates from stratospheric volcanism linked to the first pulse of the Late Ordovician mass extinction.

## Introduction

The Late Ordovician mass extinction (LOME), which eliminated ~85% of marine species globally, has been ranked as the second most severe Phanerozoic biotic crisis^1,2^. The LOME occurred in two pulses, one at the beginning of the Hirnantian stage, corresponding to the lower *N. extraordinarius* Zone and the other at the base of the *N. persculptus* Zone of the uppermost Hirnantian^1,2^. Global cooling and habitat loss resulting from the waxing and waning of glaciers during the Hirnantian have been traditionally held to have caused the LOME^3,4^. However, the cycles of glaciation and associated sea-level change during the Hirnantian likely were more complex than originally assumed^5–8^. Oceanic anoxia has been linked to the LOME^9–12^, however, temporal changes in global redox conditions that could synchronise with each pulse of the LOME remains elusive. The emplacement of a large igneous province (LIP) also has been hypothesised as a driver for the LOME based on mercury (Hg) enrichments^13,14^. However, the Hg enrichments in the sedimentary rocks of the Ordovician-Silurian boundary from South China have been argued to be sulphide-hosted rather than of volcanic origin, challenging the hypothesis of a volcanic driver for the LOME^15^. Here, we report sulphur isotope data (δ^34^S, Δ^33^S and Δ^36^S) of pyrite from two sedimentary successions from South China, and we use a characteristic signal of large Δ^33^S anomalies, resulting from stratospheric photochemical reactions^16–20^ to constrain the nature of volcanic eruptions during the Late Ordovician. Our results provide new evidence of linking stratospheric volcanic eruptions and environmental deterioration to the LOME.

## Sampling and results

### Geological setting and stratigraphy

**Fig. 1:**
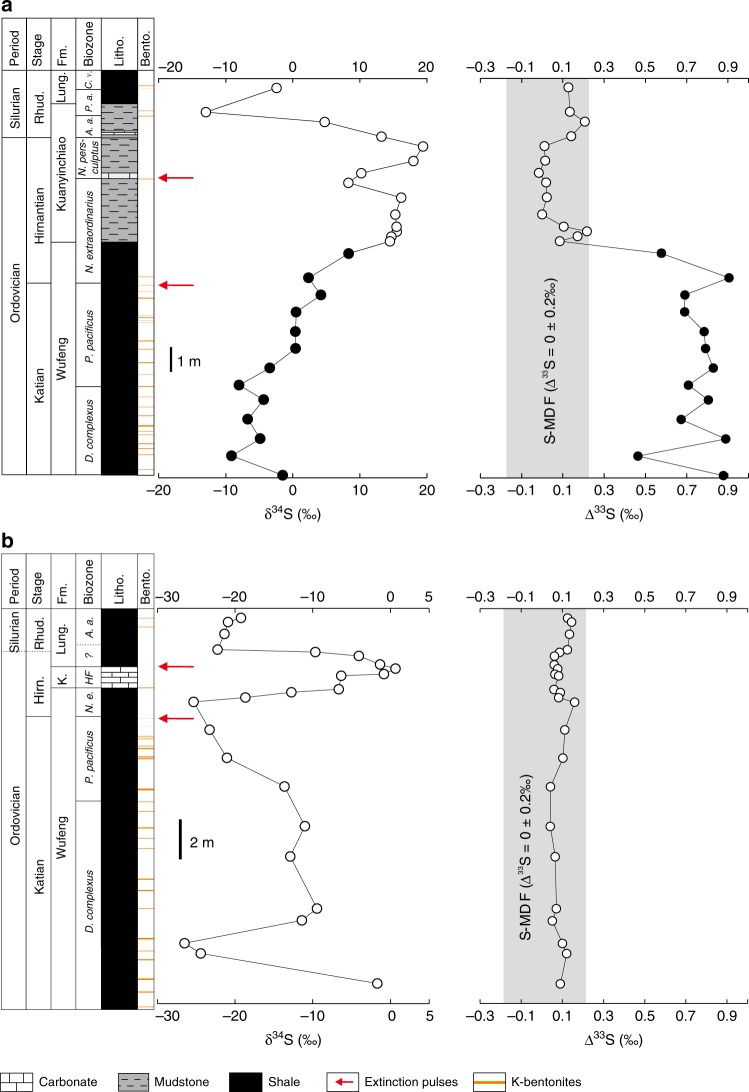
δ^34^S and Δ^33^S data for the Honghuayuan and XY5 sections. **a** Honghuayuan section. **b** XY5 core. K-bentonites are based on ref. ^24^. Filled black circles indicate S-isotope mass-independent fractionation (S-MIF), and grey fields denote Δ^33^S = 0 ± 0.2‰ representing the traditional limit of S-isotope mass-dependent fractionation (S-MDF)^27^. Analytical errors are smaller than the symbol sizes for all data reported in this study. *N.e.: Normalograptus extraordinarius*, *A.a.: Akidograptus ascensus*, *P.a.: Parakidograptus acuminatus*, *C.v.: Cystograptus vesiculosus*, Lung.: Lungmachi, K.: Kuanyinchiao, Hirn.: Hirnantian, *HF*: *Hirnantian Fauna*, Rhud.: Rhuddanian, Fm.: Formation, Litho.: Lithology, Bento.: K-bentonites.

Our samples were collected from the Honghuayuan section and a drill core (named XY5) in South China. The Honghuayuan section is a typical shallow-water section, which was deposited in an innermost sub-basin near the Dianqian uplift^21,22^ (Supplementary Fig. 1). To study isotopic changes along a paleoenvironmental gradient, we also collected the pristine core material of the deep-water section within XY5. The XY5 section was deposited near a slope setting with a likely water depth of ca. 100–200 m based on the assemblage of benthic invertebrate fossils^23^ (Supplementary Fig. 1). The Ordovician-Silurian strata in both successions consist of the Late Ordovician Wufeng Formation, Kuanyinchiao Bed and the earliest Silurian Lungmachi Formation (Fig. 1a, b). The Wufeng and Lungmachi formations are composed of black shales from which abundant and diverse graptolite fossils have been reported^22^. The Kuanyinchiao Bed is composed mainly of dark brown mudstone intercalated with thin carbonate lenses that preserve abundant shelly fossils. In both areas, numerous K-bentonite beds with thickness of 1–1.5 cm but many are <3 mm, have been found from *D. complexus* to upper *P. pacificus* Zone, indicate volcanic eruptions during the Late Ordovician in South China^24,25^ (Fig. 1a, b). The biostratigraphy of the two successions has been well established and they record the two pulses of the LOME that can be correlated regionally and globally^22^ (Fig. 1a, b).

### Multiple sulphur isotope data

**Fig. 2:**
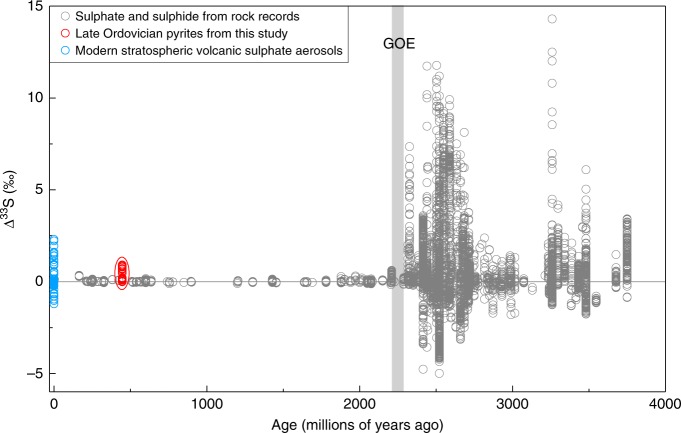
Summary of Δ^33^S data over geological time. Data within the red oval are from this study, and the stratospheric volcanic sulphate data (blue circles) are from refs. ^17,19,45,46^, and the rock record data (grey circles) are compiled from refs. ^27,28,30,31,44,47^. GOE: Great Oxidation Event.

We measured quadruple S-isotope compositions (δ^33^S, δ^34^S and δ^36^S) of pyrite from the Honghuayuan section and the XY5 core. The multiple sulphur isotope data are reported using conventional delta notation: δ^3x^S = 1000 × ((^3x^S/^32^S)_sample_/(^3x^S/^32^S)_reference_ − 1), where 3x = 33, 34, or 36. Capital delta notation is defined to describe relationships involving ^33^S and ^36^S: Δ^33^S = δ^33^S − 1000 × ((1 + δ^34^S/1000)^0.515^ − 1), Δ^36^S = δ^36^S − 1000 × ((1 + δ^34^S/1000)^1.90^  − 1). The Δ^33^S and Δ^36^S are defined with exponents of 0.515 and 1.90 to approximate the deviation from single step low-temperature equilibrium exchange reactions^26,27^. Delta and capital delta values are given in unit of per mille (‰). Figures 1–2 present δ^34^S and Δ^33^S data of pyrite from the studied successions (full analytical data of the samples and reference materials available as Supplementary Tables 1–3).

At Honghuayuan, the δ^34^S of pyrite ranges from −9.1‰ to 4.2‰ in the Katian strata and is followed by a large positive excursion of ~17‰ during the Hirnantian Stage (Fig. 1a). The δ^34^S then returns to the pre-excursion baseline value in the Rhuddanian strata (Fig. 1a). Likewise, the δ^34^S from Katian strata of the XY5 core varies between −26.5‰ and −1.7‰ and is followed by a large positive δ^34^S excursion of ~26‰ during the Hirnantian Stage (Fig. 1b). Higher in the stratigraphic section, the δ^34^S of the pyrite returns to the pre-excursion sulphur isotope composition with a narrow range of −21.4‰ to −19.3‰ in the Rhuddanian strata (Fig. 1b).

At Honghuayuan, the Δ^33^S data of the Wufeng Formation exhibit positive values varying between 0.46‰ and 0.91‰ with an average of 0.75‰ with Δ^36^S values from −0.28‰ to 0.11‰ (Fig. 1a; Supplementary Table 1). These unusually positive Δ^33^S are similar in magnitude to the S-isotope mass-independent fractionation (S-MIF) data prior to the great oxidation event (GOE) about 2.3 Gyr ago (Fig. 2), which were likely produced by the photodissociation of S-bearing gases by short wavelength ultraviolet (UV) rays in an oxygen poor atmosphere^27–32^. Coincident with the disappearance of the K-bentonite beds, the S-MIF signals disappear and the Δ^33^S varies little between −0.02‰ and 0.22‰ for the rest of the Honghuayuan section with Δ^36^S values ranging from −1.03‰ to 0.06‰ (Fig. 1a; Supplementary Table 1). However, unlike the Δ^33^S data from Honghuayuan, none of the XY5 core samples shows a signal of S-MIF; all pyrite samples from the XY5 core exhibit S-isotope mass-dependent fractionation (S-MDF) signals with the Δ^33^S ranging from 0.04‰ to 0.16‰ with an average of 0.09‰, corresponding with Δ^36^S values ranging from −0.95‰ to −0.18‰ (Fig. 1b; Supplementary Table 2).

## Discussion

### Origin of the sulphur isotope mass-independent fractionation signal

Almost all physical, chemical and biological processes fractionate S-isotopes by the relative mass differences of each isotope, producing Δ^33^S values that are near-zero^27^. Sulphur photochemistry is one of the few processes that produces mass-independent fractionation, yielding non-zero Δ^33^S values^16,29,30^. As such, S-isotopes have been successfully used in polar snow and ice cores to distinguish between stratospheric and tropospheric volcanic eruptions^16–20^. In the stratosphere, at or above the ozone layer, the sulphur ejected from volcanic emissions will be exposed to UV radiation, acquiring a S-MIF signal with non-zero Δ^33^S value^16–20,29,30^.

The Δ^33^S data varying between 0.46‰ and 0.91‰ of the Wufeng Formation at Honghuayuan are far too large to be generated by S-MDF processes alone^28,30,33^. Two other possibilities could be considered; weathering input from Archean rocks that have a non-zero Δ^33^S, and magnetic isotope effects (MIE) during thermochemical sulphate reduction (TSR). Weathering input of Archean-age sulphur minerals with S-MIF is an unlikely mechanism to produce the S-MIF signals we observe because the Precambrian basement of the inner Yangtze platform, which would comprise the rocks that could be weathered during the Late Ordovician, consists exclusively of Neoproterozoic lithologies younger than 850 million years^34^.

**Fig. 3:**
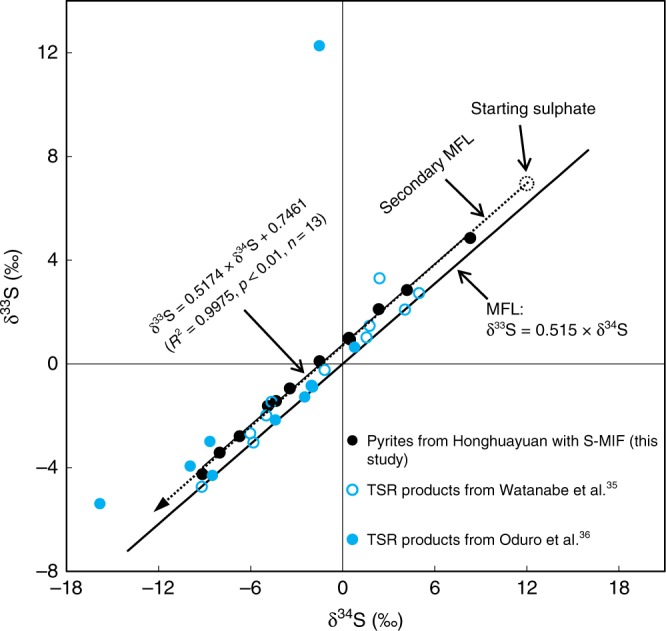
δ^34^S and δ^33^S for Honghuayuan and thermochemical sulphate reduction experiments. Filled black circles represent the S-MIF from Honghuayuan, and blue circles are the experimental data from refs. ^35,36^. The dotted line illustrates the fractionation associated with microbial reduction of sulphate with a S-MIF composition (open dotted circle) that fits all pyrites with positive Δ^33^S anomalies from Honghuayuan. MFL: mass fractionation line, S-MIF: S-isotope mass-independent fractionation, TSR: thermochemical sulphate reduction.

Experiments have shown that MIE during TSR can impart a non-zero S-MIF signature^35,36^; however, we suggest that this process is an unlikely source for our observation of S-MIF from Honghuayuan. As shown in Fig. 3, a plot of δ^34^S–δ^33^S for all pyrites that have a non-zero S-MIF at Honghuayuan follows a highly-correlated array: δ^33^S = 0.5174 × δ^34^S + 0.7461 (*R*^2^ = 0.9975, *p* < 0.01, *n* = 13). This array parallels the mass fractionation line (MFL), indicating that the mass-dependent process of microbial sulphate reduction (MSR), which would produce sulphide to form pyrite, started from a sulphate pool with a S-MIF composition (Fig. 3). In contrast, experimentally produced S-MIF by TSR often exhibits a scattered δ^34^S–δ^33^S, with little correlation^35,36^ (Fig. 3). The equivalent vitrinite reflectance (Ro) for Honghuayuan samples is low with an average of ~0.7% (Supplementary Table 4), implying that these sedimentary rocks are thermally immature and have had a maximum burial temperature <110°C (estimated from the equation in ref. ^37^), which is well below the minimum temperature for TSR in both experimental and natural conditions (~150°C^35,36^ and ~120 °C^38^, respectively). Therefore, the Ro data indicate that TSR could not have occurred in Honghuayuan samples we measured.

In addition, multiple S-isotope data from thermally mature oil shales and organic carbon-enriched sedimentary rocks with burial temperature higher than 200°C still show low δ^34^S of pyrite or organic-bound sulphide with mass-dependent Δ^33^S signals ranging from −0.055‰ to 0.073‰; these have been attributed to retain the sulphur isotopic composition acquired during MSR and/or microbial sulphur disproportionation^39,40^. Therefore, although these organic carbon-enriched and pyrite-bearing sedimentary rocks may have reached the temperature where TSR may occur, there is no isotopic evidence that TSR did occur and produce S-MIF signals. Moreover, sulphide produced by TSR in experimental or natural conditions routinely displays higher δ^34^S similar to or same as that of the parent sulphate, because TSR can be quantitative in converting sulphate to sulphide^41–43^. In contrast, our S-MIF data from Honghuayuan show consistently lower δ^34^S (Fig. 1a), further hinting at a bacteriogenic origin of the sulphide that then converted to pyrite.

**Fig. 4:**
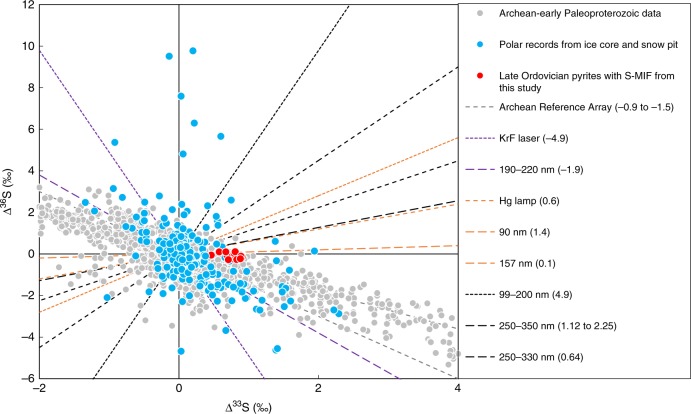
Mass-independent S-isotope effects observed in rock and polar record. The Archean-early Paleoproterozoic rock record (filled grey circles) are compiled from refs. ^27,28,30,31,44,47^, and the polar record from ice core and snow pits (filled blue circles) are from refs. ^16–18,45,46,49^. The Late Ordovician rock record (filled red circles) are from this study. The two dotted grey lines illustrate the Archean Reference Array (Δ^36^S/Δ^33^S = −0.9 to −1.5) and the Δ^36^S/Δ^33^S slopes of photochemistry experiments are compiled from refs. ^44,47^, and references therein.

We note that our S-MIF data show limited Δ^36^S variation, which does not display a statistically distinct Δ^36^S/Δ^33^S slope of −0.9 to −1.5 like most of the Archean-early Paleoproterozoic data^29,44^ or negatively correlated Δ^36^S/Δ^33^S from ice core data^16–18,45,46^ (Fig. 4). This may be due to the limited number of multiple S-isotope analysis as our S-MIF data were measured from a single section. There are Archean-early Paleoproterozoic multiple S-isotopic data that show marked Δ^33^S anomalies but with limited Δ^36^S variation, like the S-MIF at Honghuayuan (Fig. 4). Though the exact underlying mechanisms to produce the S-MIF are debated, all the Archean-early Paleoproterozoic and modern stratospheric volcanic sulphate data with large Δ^33^S anomalies have been ultimately linked to photochemical reactions^16–20,28,44–47^. There is little reason to assume that this would not be the case for our Late Ordovician S-MIF. Photochemical experiments have shown that Δ^33^S–Δ^36^S patterns are highly dependent on photochemical conditions, yielding various Δ^36^S/Δ^33^S slopes from −4.9 to 4.9 (Fig. 4). The large Δ^33^S anomalies with or without distinct relationships with Δ^36^S suggest that the volcano-atmosphere interaction in the Late Ordovician was possibly different than today, or that they might also reflect changing physical and chemical pathways for transporting and production of aerosol sulphate in the atmosphere.

Collectively, we find little evidence of TSR at Honghuayuan, and our data are inconsistent with the isotopic signals from the experimental results of TSR. There is no S-MIF example produced by TSR alone from rock record that is comparable to the S-MIF at Honghuayuan. Rather, our large Δ^33^S anomalies are comparable to the Δ^33^S found in ice core sulphate and attributed to stratospheric volcanism, which likely resulted from photochemical reactions involving sulphur species in the stratosphere at or above the ozone layer^16–20^.

The atmospheric O_2_ during the Late Ordovician was estimated to have contained ~17–30% of present atmospheric levels^48^, which is far more than that prior to the GOE, ~2 billion years earlier^28,30^. Therefore, the interpretation for the S-MIF observed in the Honghuayuan section requires a mechanism to generate S-MIF in the presence of atmospheric oxygen. The S-MIF data observed in modern stratospheric volcanic sulphate aerosols have been attributed to photochemical reactions in the upper atmosphere^16–20,45^. These observations demonstrate that the production, transport, and preservation of S-MIF signals can occur in an oxidising atmosphere. The S-MIF signals in modern volcanic sulphate aerosols are exclusively linked with violent stratospheric eruptions that have sufficient energy to inject volcanic materials into the stratosphere at or above the ozone layer where short wavelength UV (*λ* < 300 nm) radiation is available. As such, the S-MIF has been used as an important tool to trace stratospheric eruptions in the past^16–20,46,49^.

**Fig. 5:**
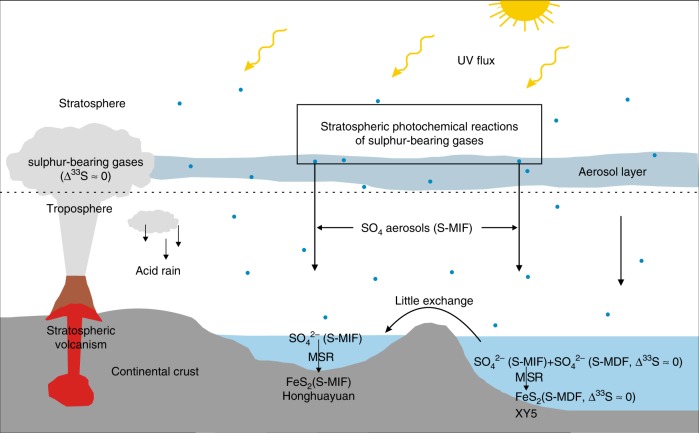
Proposed sulphur cycle for the observed isotope record. UV: ultraviolet, S-MIF: S-isotope mass-independent fractionation, S-MDF: S-isotope mass-dependent fractionation, MSR: microbial sulphate reduction.

We suggest that the Late Ordovician S-MIF signals from South China may be best explained by a similar mechanism to modern stratospheric volcanic sulphate aerosols. Our data suggest an atmospheric sulphur cycle that begins with volcanic injections of large amounts of S-bearing gases probably dominated by SO_2_ (ref. ^50^) into the stratosphere, at or above the ozone layer, where the UV flux is high (Fig. 5). The subsequent photochemical reactions of the volcanogenic SO_2_ produce mass-independently fractionated sulphate aerosols with positive Δ^33^S anomalies similar to modern stratospheric volcanic sulphate aerosols^16–20,45,46,49^.

Following the transfer of the Late Ordovician stratospheric sulphate aerosols to Earth surface environments like Honghuayuan, atmospherically derived sulphate aerosols would mix with dissolved seawater sulphate in the local environment (Fig. 5). This mixed sulphate pool may then produce sulphide through MSR, which is solely a mass-dependent process (Fig. 5). As a result, the sulphide produced through this MSR is mass-dependently isotopically fractionated with respect to the parent sulphate, with the produced sulphide depleted in ^34^S, preserved in pyrite (Figs. 3 and 5). We note that all pyrite samples with the S-MIF compositions from Honghuayuan exhibit a tight linear array parallel to the MFL, originating from microbial reduction of sulphate with a S-MIF composition (Fig. 3).

**Fig. 6:**
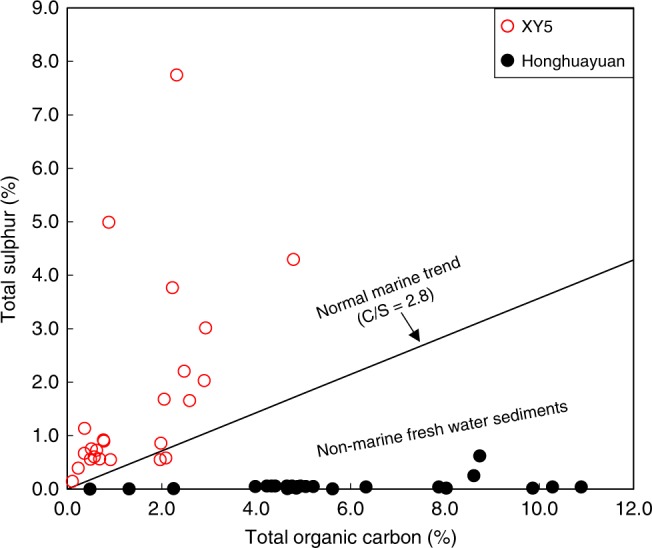
Cross-plot of total organic carbon and total sulphur. Filled black circles are from Honghuayuan, and red circles are from XY5. Black solid line represents the typical C/S ratio of ~2.8 for normal marine sediments^51^.

In contrast to the Honghuayuan section, none of pyrite samples from the XY5 core exhibit a S-MIF composition. Rather, they show limited Δ^33^S variation from 0.04‰ to 0.16‰, suggesting they result from S-MDF process, even though the K-bentonite beds are widespread in both areas^24^ (Fig. 1a, b). The significant differences in the S-isotopic compositions may reflect the difference in the depositional environment between the Honghuayuan section and that in the XY5 core. The Honghuayuan section was deposited in an innermost sub-basin near to land, where water exchange with the open ocean was quite restricted during the Late Ordovician^21^ (Fig. 5). The Honghuayuan section samples contain extremely low total sulphur content with an average of 0.07% and TS/TOC (total sulphur/total organic carbon) ratios with an average of 0.01 that are similar to those found in typical freshwater environments^51^, which usually contain orders-of-magnitude less sulphate than in normal marine environments (Fig. 6). The extremely low TS/TOC in the Honghuayuan section is consistent with our hypothesis that the major dissolved sulphate in this area was supplied atmospherically. By contrast, the XY5 core section was deposited in a relatively deep-water environment and the XY5 core samples are characterised by high total sulphur content of up to 7.75% and TS/TOC ratios of up to 5.67 that are consistent with marine conditions, which typically contains much higher sulphate than freshwater environment (Fig. 6). We suggest that the relatively high sulphur concentration and large pool of dissolved seawater sulphate where the XY5 core was deposited may have isotopically homogenised the atmospherically derived sulphate, producing sulphide with S-MDF compositions as we observe (Fig. 1b).

### Implications for the Late Ordovician mass extinction

The presence of stratospheric volcano-generated S-MIF data in the Late Ordovician sedimentary rocks from South China may provide new insight into the causes of the LOME. The stratospheric eruptions may have much more severe and prolonged impacts on atmospheric and ocean chemistry, terrestrial environments, and climate change than tropospheric eruptions^52–55^. We note that, based on Hg abundances and Hg/TOC ratios, a LIP event has been hypothesised as a driver for the LOME^13,14^. However, Hg enrichments were interpreted to have been sulphide-hosted rather than of volcanic origin, which is against the causal linkage between the LOME and volcanism^15^. Also, there is a lack of geological evidence for a Late Katian-Hirnantian LIP^13^ and the nature of the volcanic eruptions such as the explosivity and specifically, if the volcanic plumes penetrated the stratosphere remains poorly constrained. The nature of volcanic eruptions is critical because volcanic sulphur-induced climate change such as cooling or sulphuric acid-driven ocean acidification cannot cause a profound biotic crisis unless eruptions occurred with high intensity and over long durations owing to the buffering capacity of the Earth system^56^.

In this study, the S-MIF data from South China provide evidence for stratospheric volcanic eruptions during the Late Ordovician that were coincident with the first pulse of the LOME (Fig. 1a). Kill mechanisms and environmental perturbations stemming directly from the stratospheric volcanic eruptions may include sulphur-induced climate cooling, acid rain and ocean acidification from emission of SO_2_ and CO_2_, toxic metal poisoning, and anoxia associated with release of CO_2_ and other greenhouse gases as well as enhanced delivery nutrients to the ocean^52–58^. Sulphur species (mainly SO_2_, H_2_S, COS) are the primary volcanic volatiles known to impact climate directly, as their oxidation to H_2_SO_4_ in the atmosphere forms sulphate aerosols that disturb the atmospheric radiation balance^52–55^. In the troposphere, volcanogenic sulphur is rapidly oxidised to sulphate and washed out of the atmosphere^52–54^. The climate perturbations by the emission of sulphur are greatest when sulphur species are ejected into the stratosphere, where they react mainly with OH and H_2_O to form sulphate aerosols^52–55^. The stratospheric sulphate aerosols can play a critical role in Earth’s heat budget because they may backscatter incoming short-wave solar radiation and absorb outgoing long-wave radiation, leading to global cooling at the Earth’s surface^52–55^. Though climate cooling resulting from modern individual stratospheric volcanic eruptions may be relatively short, explosive stratospheric volcanic eruptions in the geological past could have prolonged and profound climatic ramification^59^. The S-MIF data we observe suggest that explosive and sustained stratospheric volcanic eruptions during the Late Ordovician could have potentially contributed to the icehouse conditions, coupled with *p*CO_2_ drawdown through enhanced silicate weathering and organic carbon burial^60,61^, though much remains enigmatic on the climatic feedback of particularly the early Hirnantian. Additional kill mechanisms for the LOME may be linked to expansion of oceanic anoxia, which has been inferred from isotopic and elemental proxies coincident with the LOME^9–12^. Hence, our S-isotopic data suggest that stratospheric volcanic eruptions during the Late Ordovician may have contributed to the synergetic environmental deteriorations such as global climatic perturbations from volcanic sulphur and CO_2_ release, ocean anoxia and acidification, and ecosystem poisoning, which may have driven the first pulse of the LOME. However, we find no evidence of the S-MIF signals during the second pulse of the LOME at Honghuayuan (Fig. 1a), suggesting little role of the stratospheric volcanism in the second pulse of the LOME.

Numerous K-bentonites beds of Ordovician-Silurian age have been identified worldwide, which probably record intensified volcanic eruptions^62^. Integrated with well-established biostratigraphy, precise dating of each pulse of the LOME, and sedimentary facies, the quadruple sulphur isotope measurements on Ordovician-Silurian rocks, especially terrestrial sedimentary rocks, can be used to test our hypothesis and improve our understanding of global climate change and its causal relationships with the LOME. Our study demonstrates that the use of quadruple sulphur isotopes is an important tool to explore atmospheric and ocean chemistry changes related to volcanic eruptions and their potential links to the biological evolution.

## Methods

### Pyrite extraction, total sulphur and total organic carbon measurement

Pyrite was extracted with the Cr-reduction method and the sample powders were reacted with a solution of 6 n HCl and reduced chromium chloride (Cr (II)) at ~80°C. The reaction proceeded for ~2 hours to liberate hydrogen sulphide, which was driven by a flow of nitrogen gas through a water-cooled condenser and a bubbler filled with Milli-Q water, and then quantitatively precipitated as silver sulphide (Ag_2_S) by reacting with silver nitrate solution. TS and TOC were measured by a CS-902T high-frequency infra-red carbon-sulphur analyzer. The bulk and decarbonated powdered samples were weighed and combusted in an oxygen atmosphere by an induction furnace. The TS and TOC contents were determined by the infra-red detector, yielding analytical uncertainties of ±3% and ±0.5% of reported values, respectively.

### Sulphur isotopic analyses

Multiple S-isotope analyses followed the published procedures^29,33^. Ag_2_S was fluorinated with a ×10 excess of F_2_ at ~250°C overnight in a Ni reaction vessel. The product SF_6_ was isolated from the residual F_2_ into a liquid-nitrogen cooled trap, distilled at ~−110°C from condensable contaminants and then further purified by a gas chromatograph equipped with a composite molecular sieve 5 Å lead/Haysep-Q column. S-isotope compositions of the clean SF_6_ were measured using a ThermoFinnigan MAT 253 gas source mass spectrometer where the ion beams at *m/z* = 127, 128, 129 and 131 were detected simultaneously. Isotope data are reported in ‰ relative to Vienna Canyon Diablo Troilite. The uncertainties were assessed by repeated fluorination and measurements of a reference material of IAEA S1 and are estimated to be better than ±0.2‰, ±0.01‰ and ±0.2‰ for δ^34^S, Δ^33^S and Δ^36^S, respectively. The XY5 samples were measured at University of Science and Technology of China, and the Honghuayuan samples were measured at the University of Maryland (UMD).

### Inter-laboratory measurements

Three samples from Honghuayuan were re-analysed at Institut de Physique du Globe de Paris (IPGP), France using the same method of pyrite extraction, fluorination of Ag_2_S and measurement of S-isotope compositions as that of UMD. As shown in Supplementary Table 3, the measurements at IPGP reproduce reasonably well the S-isotope values from UMD.

### Equivalent vitrinite reflectance

The samples were prepared to be a polished petrographic mount with a flat and smooth surface suitable for microscopy. The maceral observation and random graptolite reflectance measurements were conducted using a Leica reflected light microscope equipped with a CRAIC microscope photometer. The reflectance measurements were taken at 546 nm wavelength in oil immersion, after calibrating the microscope against two internal standards. A total of ~40 such measurements were performed per sample. The equivalent vitrinite reflectance (Ro) values were then calculated from the mean random graptolite reflectance (GRo) on the basis of the conversion equation: Ro = 0.8 × GRo (ref. ^63^).

## Supplementary information

Supplementary Information

## Data Availability

All data reported in this study are available in Supplementary Tables 1–4.
