# Supplementary material for: Large mass-independent sulphur isotope anomalies link stratospheric volcanism to the Late Ordovician mass extinction
Source: Nat Commun. 2020 May 8;11:2297. doi: 10.1038/s41467-020-16228-2 (PMC7210970; doi:10.1038/s41467-020-16228-2)
Supplement: Supplementary file 1 — Supplementary Information [file 41467_2020_16228_MOESM1_ESM.pdf]

SUPPLEMENTARY INFORMATION

Large mass-independent sulphur isotope anomalies link stratospheric volcanism to the Late Ordovician mass extinction

Dongping Hu[1], Menghan Li[1], Xiaolin Zhang[1], Alexandra V. Turchyn[2], Yizhe Gong[1], Yanan Shen[1]✉

[1]School of Earth and Space Sciences, University of Science and Technology of China, Hefei 230026, China.

[2]Department of Earth Sciences, University of Cambridge, Cambridge CB2 3EQ, UK.

✉email: yashen@ustc.edu.cn

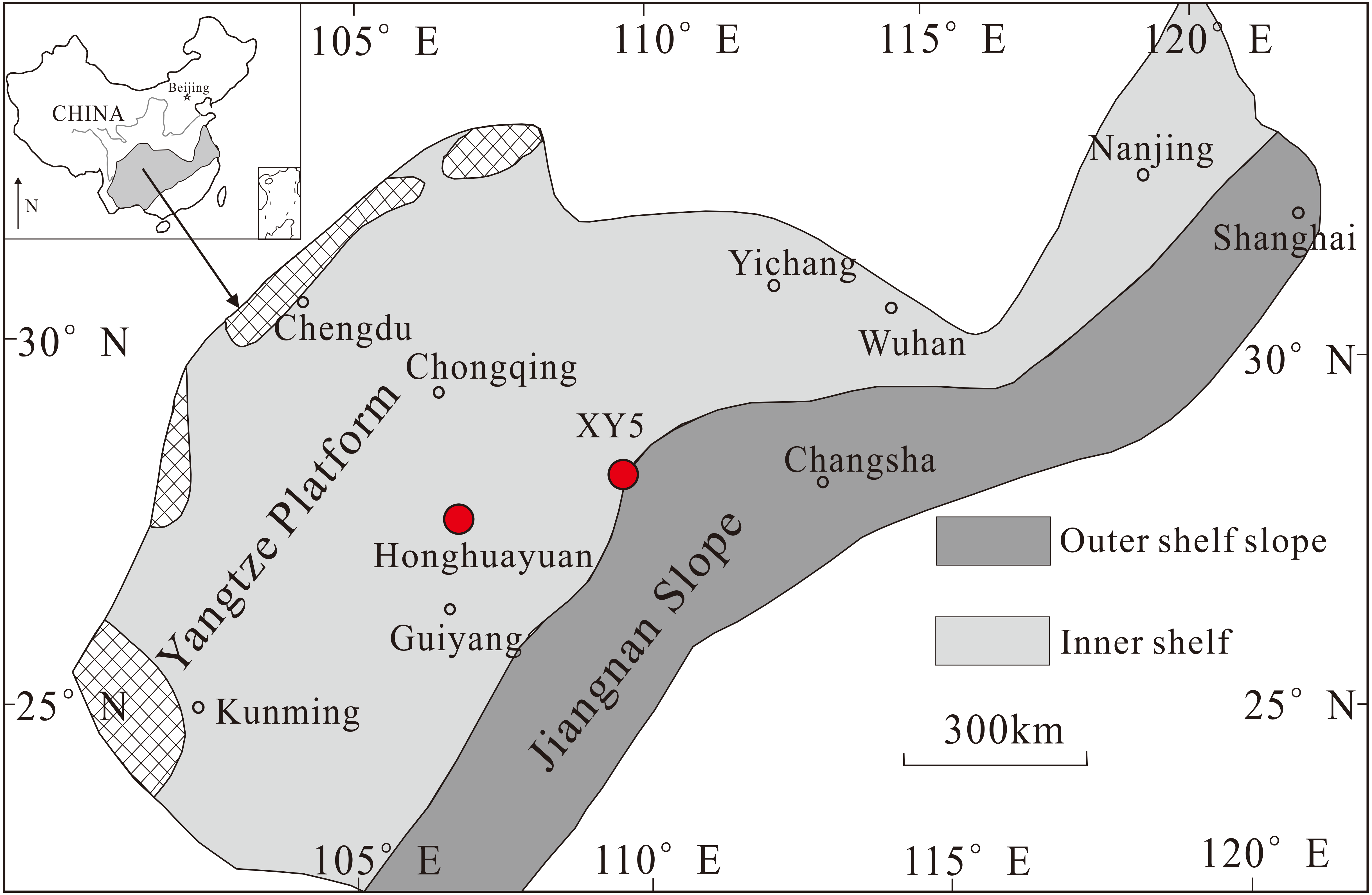

Supplementary Figure 1. Location and paleogeography map for Honghuayuan section and XY5 core (after ref. 9).

Supplementary Table 1. Geochemical data of Honghuayuan section

Sample	Formation	Stage	Lithology	$\delta^{33}S$ (‰)	$\delta^{34}S$ (‰)	$\delta^{36}S$ (‰)	$\Delta^{33}S$ (‰)	$\Delta^{36}S$ (‰)	TOC (%)	TS (%)	Height (m)
HHY-48	Lungmachi	Rhuddanian	Shale	-1.12	-2.42	-5.04	0.13	-0.46	1.31	<0.01	16
HHY-46	Kuanyinchiao	Rhuddanian	Mudstone	-6.56	-12.96	-25.51	0.13	-1.03	0.48	<0.01	15
HHY-44	Kuanyinchiao	Rhuddanian	Mudstone	2.65	4.75	8.52	0.21	-0.53	2.25	0.01	14.6
HHY-43	Kuanyinchiao	Rhuddanian	Calcareous mudstone	6.93	13.23	25.28	0.14	0.00	4.66	0.01	14
HHY-42	Kuanyinchiao	Hirnantian	Mudstone	9.96	19.41	37.02	0.01	-0.18	4.85	0.02	13.6
HHY-40	Kuanyinchiao	Hirnantian	Mudstone	9.23	17.97	34.22	0.01	-0.19	4.73	0.03	13
HHY-37	Kuanyinchiao	Hirnantian	Calcareous mudstone	5.23	10.21	19.39	-0.02	-0.09	8.03	0.02	12.5
HHY-35	Kuanyinchiao	Hirnantian	Calcareous mudstone	4.27	8.28	15.74	0.02	-0.04	8.74	0.62	12.1
HHY-32	Kuanyinchiao	Hirnantian	Mudstone	8.30	16.14	30.89	0.02	0.00	6.33	0.04	11.5
HHY-25	Kuanyinchiao	Hirnantian	Mudstone	7.84	15.28	29.08	0.00	-0.16	8.62	0.25	10.8
HHY-20	Kuanyinchiao	Hirnantian	Mudstone	8.03	15.46	29.23	0.10	-0.34	7.87	0.04	10.3
HHY-18	Kuanyinchiao	Hirnantian	Mudstone	8.17	15.50	29.35	0.22	-0.31	10.28	0.04	10.1
HHY-16	Kuanyinchiao	Hirnantian	Mudstone	7.70	14.68	28.13	0.17	0.06	10.89	0.04	9.9
HHY-14	Kuanyinchiao	Hirnantian	Mudstone	7.51	14.46	27.65	0.09	-0.01	9.86	0.02	9.7
HHY-13	Wufeng	Hirnantian	Shale	4.86	8.32	15.97	0.58	0.10	5.21	0.05	9.2
HHY-12	Wufeng	Hirnantian	Shale	2.12	2.35	4.33	0.91	-0.14	5.05	0.05	8.2
HHY-11	Wufeng	Katian	Shale	2.85	4.19	7.95	0.69	-0.03	4.76	0.06	7.5
HHY-10	Wufeng	Katian	Shale	0.94	0.49	0.86	0.69	-0.08	4.33	0.06	6.8
HHY-9	Wufeng	Katian	Shale	0.98	0.39	0.49	0.78	-0.25	4.40	0.06	6
HHY-8	Wufeng	Katian	Shale	1.01	0.42	0.82	0.79	0.03	4.23	0.06	5.3
HHY-7	Wufeng	Katian	Shale	-0.94	-3.44	-6.79	0.83	-0.27	4.65	0.06	4.5
HHY-6	Wufeng	Katian	Shale	-3.42	-8.01	-15.43	0.71	-0.28	3.98	0.05	3.8
HHY-5	Wufeng	Katian	Shale	-1.43	-4.34	-8.12	0.81	0.11	4.91	0.05	3.2
HHY-4	Wufeng	Katian	Shale	-2.79	-6.71	-12.60	0.67	0.10	4.76	0.04	2.4
HHY-3	Wufeng	Katian	Shale	-1.62	-4.86	-9.32	0.89	-0.10	4.88	0.05	1.6
HHY-2	Wufeng	Katian	Shale	-4.25	-9.13	-17.34	0.46	-0.06	5.62	0.01	0.9
HHY-1	Wufeng	Katian	Shale	0.11	-1.50	-3.07	0.88	-0.23	4.94	0.06	0.1

Supplementary Table 2. Geochemical data of XY5 core

Sample	Formation	Stage	Lithology	$\delta^{33}S$(‰)	$\delta^{34}S$(‰)	$\delta^{36}S$(‰)	$\Delta^{33}S$(‰)	$\Delta^{36}S$(‰)	TOC(%)	TS(%)	Depth(m)
XY5-75	Lungmachi	Rhuddanian	Shale	-9.83	-19.25	-37.11	0.13	-0.85	0.57	0.60	19.35
XY5-74	Lungmachi	Rhuddanian	Shale	-10.68	-20.91	-40.20	0.14	-0.84	0.77	0.90	19.15
XY5-71	Lungmachi	Rhuddanian	Shale	-10.93	-21.36	-41.15	0.13	-0.95	0.69	0.56	18.55
XY5-62	Lungmachi	Hirnantian	Shale	-11.39	-22.24	-42.69	0.12	-0.87	0.37	1.13	17.78
XY5-59	Lungmachi	Hirnantian	Shale	-4.90	-9.67	-18.91	0.09	-0.62	0.88	4.99	17.65
XY5-55	Lungmachi	Hirnantian	Shale	-2.03	-4.06	-8.29	0.06	-0.58	0.23	0.39	17.46
XY5-50	Lungmachi	Hirnantian	Shale	-0.63	-1.34	-3.01	0.06	-0.47	0.36	0.67	17.03
XY5-48	Kuanyinchiao	Hirnantian	Limestone	0.41	0.65	0.74	0.08	-0.50	0.49	0.56	16.83
XY5-46	Kuanyinchiao	Hirnantian	Limestone	-0.38	-0.87	-2.05	0.06	-0.40	0.51	0.75	16.56
XY5-45	Kuanyinchiao	Hirnantian	Limestone	-3.19	-6.35	-12.39	0.08	-0.36	0.62	0.73	16.48
XY5-40	Wufeng	Hirnantian	Shale	-3.37	-6.65	-12.99	0.06	-0.39	2.93	3.02	15.8
XY5-39	Wufeng	Hirnantian	Shale	-6.50	-12.75	-24.78	0.09	-0.70	4.79	4.30	15.65
XY5-38	Wufeng	Hirnantian	Shale	-9.58	-18.68	-35.65	0.08	-0.45	2.90	2.03	15.4
XY5-37	Wufeng	Hirnantian	Shale	-12.97	-25.34	-48.02	0.16	-0.42	2.48	2.20	15.17
XY5-28	Wufeng	Katian	Shale	-11.94	-23.27	-44.52	0.11	-0.77	2.23	3.77	13.8
XY5-25	Wufeng	Katian	Shale	-10.79	-21.04	-40.21	0.10	-0.61	2.59	1.66	12.4
XY5-22	Wufeng	Katian	Shale	-7.01	-13.65	-26.34	0.04	-0.57	2.05	1.68	10.98
XY5-18	Wufeng	Katian	Shale	-5.65	-11.02	-21.07	0.04	-0.23	1.98	0.86	9
XY5-15	Wufeng	Katian	Shale	-6.60	-12.89	-24.85	0.07	-0.49	2.09	0.58	7.5
XY5-10	Wufeng	Katian	Shale	-4.81	-9.46	-18.52	0.07	-0.62	0.91	0.55	4.92
XY5-9	Wufeng	Katian	Shale	-5.83	-11.39	-21.72	0.05	-0.18	0.76	0.92	4.32
XY5-7	Wufeng	Katian	Shale	-13.63	-26.48	-50.15	0.10	-0.43	1.97	0.55	3.19
XY5-6	Wufeng	Katian	Shale	-12.53	-24.41	-46.42	0.12	-0.54	2.31	7.75	2.69
XY5-3	Wufeng	Katian	Shale	-0.79	-1.71	NA	0.09	NA	0.11	0.15	1.19

Supplementary Table 3. Interlaboratory S-isotopic measurement comparisons for 3 samples from Honghuayuan

Sample	$\delta^{34}S$(‰)	$\Delta^{33}S$(‰)	$\Delta^{36}S$(‰)	Lab
HHY-37	10.21	-0.02	-0.09	UMD
HHY-37	11.72	0.03	-0.17	IPGP
HHY-2	-9.13	0.46	-0.06	UMD
HHY-2	-9.30	0.42	-0.27	UMD
HHY-2	-8.23	0.43	-0.21	IPGP
HHY-1	-1.50	0.88	-0.23	UMD
HHY-1	-1.53	0.85	0.03	IPGP

UMD: University of Maryland; IPGP: Institut de Physique du Globe de Paris

Supplementary Table 4. Equivalent vitrinite reflectance data of Honghuayuan section

Sample	Formation	Stage	Lithology	Equivalent vitrinite reflectance (Ro)(%)	Height (m)
HHY-48	Lungmachi	Rhuddanian	Shale	0.74	16
HHY-46	Kuanyinchiao	Rhuddanian	Mudstone	0.66	15
HHY-13	Wufeng	Hirnantian	Shale	0.76	9.2
HHY-11	Wufeng	Katian	Shale	0.53	7.5
HHY-10	Wufeng	Katian	Shale	0.73	6.8
HHY-9	Wufeng	Katian	Shale	0.80	6
HHY-8	Wufeng	Katian	Shale	0.94	5.3
HHY-6	Wufeng	Katian	Shale	0.66	3.8
HHY-5	Wufeng	Katian	Shale	0.80	3.2
HHY-4	Wufeng	Katian	Shale	0.66	2.4
HHY-2	Wufeng	Katian	Shale	0.62	0.9
HHY-1	Wufeng	Katian	Shale	0.56	0.1
